# Predicting disability retirement among Abu Dhabi police using multiple measure of sickness absence

**DOI:** 10.1186/s12889-022-13713-9

**Published:** 2022-07-09

**Authors:** Faisal Almurbahani Alkaabi

**Affiliations:** Faculty of Resilience, Rabdan Academy, Abu Dhabi, United Arab Emirates

**Keywords:** Abu Dhabi, Police, Sickness absence, Disability retirement, Case–control design

## Abstract

**Background:**

Disability retirement has been investigated in the last two decades using predictors such as measures of sickness absence, psychological, social, and organizational work factors. The impact of various health-related and sickness measures on disability retirement across various occupational group reveal a significant relation. However, current literature lacks understanding in police personnel.

**Methods:**

This study examines the roles of demographic and measures of sickness absence on disability retirement among police personnel in Abu Dhabi, UAE. The case–control design was used to predict disability retirement wherein controls were matched with cases according to age and gender from those who worked in the same administration as the case at baseline, to reduce the possible confounding influence of these variables. Conditional logistic regression models were used determine the odds-ratio of various measures of sickness absence in predicting disability retirement.

**Results:**

Results indicate that increased number of spells, and number of days of sickness absence can predict disability retirements among police personnel in the UAE. Results indicate that odds ratios for disability retirement for the total exposure period increased from 1.76 (95% CI = 1.42-2.20) for spells of 4-7d to 2.47 (95%CI = 1.79-3.40) for spells of > 4 weeks. When compared with their married counterparts, non-married police employees had a statistically significant increase in odds of disability retirement of almost three fold (OR = 2.93, 95% CI = 1.55-5.56). Non-field and field police officers, on the other hand, had significantly reduced odds of disability retirement compared with admin/supportive staff (OR = 0.43 and 0.28 with 95% CI = 0.19-0.96 and 0.13-0.61 respectively). Odds ratios of disability retirement at end of the exposure period for the matching variables with those obtained after additionally adjusting for all demographic variables (model b), namely, marital status, occupation, employment grade and type, and educational level. The odds ratios of disability retirement remained significantly raised for the total number of days of sickness absence and for the number of spells of sickness absence for all spell types.

**Conclusions:**

Recommendation to reduce the number of future disability retirements among Abu Dhabi Police include structured problem-solving process addressed through stepwise meetings between the line-managers and the employee.

## Background

Work-related disability retirement in working-aged people, especially due to long-term sickness absence has been a subject of investigation for over two decades now [1, 2]. Avoiding disability retirement has become a great public health concern for many countries worldwide. This is because of the negative effect it has on the quality of life of those granted disability retirement and their families [3] as well as the extensive utilization of healthcare resources irrespective of causes of morbidity [4, 5]. For instance, owing to sickness absence, 141.4 million working days were lost in the UK in 2018,^1^ equating to 4.3 days lost per worker [6].

Studies showed that sickness absence (using various measures) is a predictor of future disability retirement. These measures include the number of days of sickness absence [7–9] average length of sickness absence spells [3, 10] and sickness absence due to certain diseases such as mental illness [9, 11], rheumatoid arthritis and fibromyalgia [8, 12]. The results with respect to number of spells of sickness absence and disability retirement were mixed: Borg et al. [3] did not find an association while Varga et al. [9] found it to be predictive, in particular for individuals with long term sickness absence spell (classified in this study as ≥ 4 days).

Sickness absence in stressful jobs such as policing, fire service personnel, and emergency responders is reported to be high [13]. Police duties include response to emergency calls, crime prevention and investigation within their jurisdiction. The occupational hazards that make police distinct from other professions include exposure to harassment, violence, threats, instances of shiftwork, and in some cases discrimination [14–16]. Exposure to such stressful situations over a prolonged period cause physical and mental illness [17] thus increasing the possibility of disability retirement among police personnel [18].

Findings from previous studies that investigated the relationship between sickness absence and disability retirement are limited for four main reasons. First, most of the previous studies may lack generalizability as they were conducted in Scandinavian countries where the labor market has low wage inequality, generous social insurance schemes and high employment rate [19]. Secondly, despite the high number of reported sickness absences, occupational injuries and early retirement in the police force [18, 20, 21], none of the previous studies investigated the relationship between sickness absence and disability retirement in this occupational group. Thirdly, few studies have analyzed number of sickness spells to investigate disability retirement [9, 22] or average length of sickness absence spells [3, 5, 10] as a risk marker for future disability retirement. Therefore, more research needs to be carried out to provide further insights into these relationships.

Therefore, this study examines sickness absence as a predictor of disability retirement using various measures of sickness absence including the number of spells of sickness absence, the total number of days of sickness absence and the average length of sickness absence spells. This study is the first to investigate this relationship utilizing data from the police force for the United Arab Emirates (UAE). The study also aims to determine which measure of sickness absence is the strongest marker for disability retirement. The outcome of this study will help identify the key reasons predictors of disability retirement among police personnel in the UAE and propose strategies to balance prevention of income loss and mitigate the substantial costs to society and the government due to loss of workforce [23].

## Linking absence to retirement

### Sickness absence in the police

Police work involves exposure to certain occupational hazards which might endanger the health of officers, such as handling law enforcement, exposure to violent situations, negative attitudes or even threats from the public and uncomfortable work schedules. Thus, and due to the complexity of their operational activities and working environment, the rate of sickness absences and occupational injuries in organisations such as the police, fire fighting and other emergency response services is higher than in other organisations [18, 24, 25]. Arokosi et al. [26] reported a significantly higher risk of sickness absence in the police than in hairdressers, loggers and female farmers. This higher risk in the police was also supported by Ferrari et al. [27], who showed an increase in risk for the police by 75% for short term sickness absence (2–7 days) and 84% for long term (≥ 27 days) compared with administrators. Other instances of exposure which have been linked to an increasing risk of sickness absence in the police significantly include driving a car for long periods as part of the job [28], police stress [29], career progression frustration (only in females), exposure to major manhunt [30] and passive smoking (only in males).

As for work exposures and sickness absence, studies on sickness absence in the police have shown that previous studies focused mainly on evaluating the effect of the exposure to violence, passive smoking and discrimination on sickness absence but did not examine other more common physical work exposures such as ergonomics, noise, vibration and repetitive movements [21]. On the other hand, only few studies evaluated the influence of psychosocial work environment on sickness absence using the job control, demand and support model and the effort/reward imbalance model [13]. However, generalizing the findings of this study is considered to be difficult for many reasons. For example, the study had a small sample size (*n* = 290) and only included police officers who were involved to maintain law and order during a major event (the Genoa G8 Summit in 2009). The sample also included mainly senior officers with not less than six years of experience. The study was carried out in Italy and the findings could still be generalized to other police departments, particularly in Europe, because labor laws and social contexts are relatively similar. However, generalizing Magnavita & Garbarino’s [13] findings to police departments outside the European continent (in the Middle East for example) is difficult at best.

Few studies investigated the influence of work-related exposures such as job control/demand on police. These work-related factors predicted sickness absence in other occupational groups such as nursing staff [31, 32] and factory employees [33–35]. A recent review of literature on occupational hazards and injuries among police officers [36] reported work-related injuries, physical hazard such as noise induced hearing loss, chemical and biological hazards due to exposure to chemical substances, blood borne diseases, and ergonomic hazards such as musculoskeletal disorders from driving long distances and lifting heavy objects. While the reviewed studies indicate the link of such hazards and injuries in sickness leave, its association with disability retirement remains vague.

Furthermore, despite the high rate of disability retirement and sickness absence in the police forces [21, 37] studies have been conducted to evaluate sickness absence and the risk of disability retirement in the police. Moreover, the literature on the relationship between sickness absence and disability retirement showed that most of the previous studies have adjusted for age and gender and only one study adjusted additionally for other sickness absence measures [38].

### Relating sickness absence to disability retirement

On the other hand, the most common measures of sickness absence used in most studies were the total number of days of sickness absence and the medical diagnosis of sickness absence. Total number of days of sickness absence generally predicted disability retirement [5]. The risk of disability retirement increased in individuals with sickness absence due to mental, musculoskeletal, nervous and gastrointestinal diseases but the findings regarding those with sickness absence due to cardiovascular and respiratory diseases were mixed [8].

Other less commonly used sickness absence measures include the number of sickness absence spells and the average length of sickness absence spell. The findings of the two studies which evaluated the association using the number of sickness absence spells were contradictory [3, 9, 22] while the average length of sickness absence spell was a predictor of disability retirement [3, 5]. More studies are needed to provide further insights into the relationship between the number of sickness absence spells and the average length of sickness absence spells and the risk of disability retirement. Furthermore, previous studies found that the risk of disability retirement increases in females and older individuals [39–41]. Finally, although many studies investigated the influence of duration of sickness absence on disability retirement, none of the previous studies quantified the effect to describe the number of days of sickness absence needed to obtain a certain probability of disability retirement [10].

Studies investigating long-term sickness as a predictor of disability retirement have also looked at occupational class differences among these retirees. For instance, a study by Salonen et al. [22] used a cohort design and reported that increase in length of sickness absence increased the risk of disability retirement in all occupational group. Job strain, measured as high level of demand and low level of control, has been identified as a significant predictor of disability retirement [42]. One particular occupational group that suffers from such job strain are the law-enforcement agencies. The current literature has given little attention to this group, especially in the middle-eastern context. Hence this study fills an important gap in analyzing the relation between sickness absence (both short-term and long-term) and disability retirement among the police in the United Arab Emirates (UAE).

### Abu Dhabi police: sickness absence and disability retirements procedures

Abu Dhabi (AD) is the capital city of the UAE with a population of about 1.5 million [43]. Abu Dhabi Police is a part of the Ministry of Interior of the UAE and consist of six General Directorates. Each General Directorate consists of several Administrations, which are further divided into departments, branches and units. The General Directorate of Policing Operations is the largest employee recruiter and the only one that has Directorates rather than Administrations within its organizational structure. Although official statistics are unpublished, Abu Dhabi Police employs over 35,000 police officers and civilians. Therefore, evaluation of sickness absence, disability retirement and early retirement intentions in the Police force, one of UAE’s largest employee recruiters, would provide the government with insights into specific policing occupational health and safety requirements. The police in the UAE primarily constitutes of UAE nationals while non-nationals mainly work as civilians. The policies for absence and retirement vary by emirates and across public and private agencies. However, all public entities of Abu Dhabi follow the same structure to implement the sickness absence and disability retirement policy.

The regulations dictating sickness absence and disability retirement in Abu Dhabi is structured. For sickness absence, an AD police employee can visit any medical doctor requesting sickness absence. This has to be then approved by the Health Authority of Abu Dhabi (HAAD). If the requested sickness absence is less than 14 days, the absence certificate is stamped by the Medical Affairs Director of AD Police else it has to be approved by the AD Police Medical Committee which, in either case, is uploaded and registered in the Human Resource Department.

Disability Retirement can be requested by the employee, a medical doctor or the department of the employee. The AD Police Medical committee comprised of medical professionals, representatives from the Financial Directorate, the Legal Affairs Administration, and the Minister’s Office evaluates the application thoroughly for acceptance or rejection. Once approved, the request is effective immediately. At federal level, the Ministry of Health and Prevention (MOHAP) monitors the regulation of disability retirement.

## Methodology

Although a cohort design is more suitable for this type of investigation, it could not be adopted for two main reasons. First, that design may have provided indications about the general demographic characteristics of the entire Abu Dhabi Police force, which are considered to be ‘sensitive’. Secondly, sickness absence recording only commenced in 2010 and the primary outcome ‘disability retirement’ in this study was rare (an average of 26 disability retirement cases per year between 2010 and 2012 from a total of 35,000 employees). Thus, a case control design was regarded as the most feasible design to implement in the short time available, as pointed out by Hennekens et al., [44] and Hennesy et al. [45]. Controls were matched with cases according to age and gender from those who worked in the same Administration as the case at baseline, to reduce the possible confounding influence of these variables.

### Dependent variable: disability retirement

The primary outcome for this part of the research is retirement due to disability or ill-health. In the Abu Dhabi Police, the request for disability retirement is made by the employee, a medical doctor or an employee’s department. The application is received by the Abu Dhabi Police Medical Committee, which comprises medical professionals, representatives from the Financial Directorate, the Legal Affairs Administration and the Minister’s Office.

The committee meets every month and evaluates applications after a thorough examination of employees’ medical files and work performance. Each employee has the right to appeal through the Abu Dhabi Police Legal Affairs Administration, whether the request is accepted or rejected. If medical retirement is granted, it is considered effective immediately.

All cases of ill health retirement in the Abu Dhabi Police between 2010 and 2012 were obtained from the Human Resources Directorate. There were 78 cases of disability retirement between 2010 and 2012, representing 31, 20 and 27 cases for 2010, 2011 and 2012, respectively. The age (categorized into 19–24, 25–29, 30–34… and 60 +) and gender of each case at baseline (two years prior to retirement for each case) were then used to search for eligible controls who worked in the same administration as the case at baseline, using the electronic human resource system of the Abu Dhabi Police. When controls could not be found using these criteria, the search was expanded to look for controls within the General Directorate where the case was working at baseline. This was performed for only one of the cases of disability retirement.

In order to have a similar number of controls for each case and maintain power, it was decided that sickness absence records for five randomly selected controls (using an electronic system) per case would be selected (for those cases with six or more controls), as recommended by Rothman [46], and Hennessy et al. [45]. Sickness absence records for each set of case and matched controls for one and two years before retirement were then obtained from the system. The final records included 75 sets of cases and controls (31, 20 and 24 disability retirement cases in 2010, 2011 and 2012 respectively); three cases were excluded because they started working for the police in 2012 and were granted disability retirement in the same year (due to involvement in major accidents). There were 344 controls, an average of 4.5 controls per case.

### Independent variables

#### Demographic measures

In addition to the matching variables, the following demographic variables were extracted from Human Resources (HR) records: marital status, educational level, occupation, employment type (civilian/officer), employment grade, and years of service. Employment grade from HR records was grouped by the researcher into administrative/supportive staff, field and non-field police employees. Admin/supportive staff include correspondents, equipment technicians, typists, translators and medical staff at the police hospital. Field police officers include armed officers, investigators, crime scene officers, inspectors and police car drivers whereas non-field officers include crime telephone operators, admin officers, weapon store employees and prison guards.

#### Sickness measures

##### Sickness absence spells

Abu Dhabi Police implements a rigid system of recording of sickness absence whereby all spells of sickness, regardless of duration, are recorded in the Human Resource System. For each spell of sickness absence, details recorded include date of absence and duration of spell in days. This information was used to compute the total number of spells and overall duration in the year and total sickness absence throughout the years of service.

For each record, sickness absence was classified as long term if number of days of sickness absence (or duration) was four weeks or more, as recommended by the National Institute of Health and Care Excellence (NICE) in England [47]. As a “fit to work note” is commonly required for spells lasting 8 days or more [48], a further category for spells between 8 days and 4 weeks (defined as 28 days) was introduced in this study. In order to compare results from this study with the existing literature, two more categories of sickness absence spells were added namely, 1–3 days and 4–7 days. Thus, categories of sickness absence spells used in the study are 1-3d, 4-7d, 8-28d and spells lasting more than four weeks (> 4 weeks) (Table 1). The distribution indicates that 44% of the cases (33/75) had 11 or more spells during the two-year exposure period compared with only 10% of controls (34/344). One third of cases (26/75) had between 1 and 7 spells whereas two thirds of controls (230/344) had sickness absence spells within this range.

**Table 1:** Sickness spells by days for both disability retirement cases and controls

**Number of spells**	**Cases**		**Controls**	
	***N***	***(%)***	***N***	***(%)***
**0**	7	9.3	61	17.7
**1–3 spells**	15	20	161	46.8
**4–7 spells**	11	14.7	69	20
**8–10 spells**	9	12	19	5.5
**11–20 spells**	17	22.7	30	8.7
**21 + spells**	16	21.3	4	1.1
***Total***	75	100%	344	100%

For any spell that overlapped between one calendar year and the next, such as baseline (two years before retirement), to follow up (one year before retirement), the spell was counted in the year that it started but the overlapping sickness absence days were added to the following year. For example, if a case had a spell of 10 days’ duration starting on December 31^st^ of 2010, the spell was counted in the total number of spells for 2010 records but only one of its days was included in the duration for 2010 and the remaining 9 days in that spell were added to the duration for 2011. This concurs with procedures previously implemented in the Whitehall II study [49].

##### Number of days of sickness absence

Table 2 shows the distribution of the number of days of sickness absence in cases and controls over the two-year exposure period. Approximately one-third of the disability retirement cases had less than 40d of sickness absence and a similar percentage had 160d or more of sickness absence over the two-year exposure period. Most of the controls (90%) on the other hand, had either zero days of sickness absence (18%) or between 1 and 39d of sickness absence (72%) during the exposure period.

**Table 2:** Distribution of total sickness absence for cases and controls

Total number of days of sickness absence	Cases (*n* = 75)		Controls (*n* = 344)	
	***N***	***(%)***	***N***	***(%)***
**0**	7	9.3	61	17.7
**1–39**	19	25.3	249	72.4
**40–79**	9	12	18	5.2
**80–119**	7	9.3	4	1.2
**120–169**	6	8	1	0.3
**160–199**	7	9.3	1	0.3
**200 + **	20	26.7	10	2.91
***All durations***	75	100%	344	100%

It is notable that while about 10% of the controls took 40 or more days of sickness absence, over 65% of disability retirement cases report sickness absence of 40 or more days. This distribution indicates the relationship of number of days of sickness absence with disability retirement.

### Statistical analysis

Each set of one case and one or more matched controls was given a unique identifier (ID). Descriptive statistics using counts and percentages were used for demographic variables and means and standard deviations were used to describe number of days of sickness absence and number of sickness absence spells. The mean number of sickness absence spells (Table 3) for controls were obtained by calculating the mean for the controls in each case/control set (resulting in 75 control means). The mean of the means for controls was then calculated. This was done to reduce bias in reporting of estimates as cases did not have same number of controls.

**Table 3:** Description of demographic variables of disability retirement cases and the controls

Variable		Total Sample (*n* = 419)			
	Measure/Code	*Cases (n* = *75)*		*Controls (n* = *344)*	
		N %		N %	
Age					
18–29	1	24	32	107	31.1
30–39	2	22	29.3	119	34.6
40–49	3	14	22.7	81	23.6
50–59	4	11	14.7	34	9.9
60 +	5	1	1.3	3	0.9
Gender					
Female	1	16	21.3	66	19.2
Male	2	59	78.7	278	80.8
Marital Status					
Married	1	44	58.7	263	76.4
Non-Married	2	31	41.3	81	23.6
Admin/ supportive	1	23	30.7	57	16.6
Field Policing	2	35	46.7	210	61.1
Non-Field Policing	3	17	22.7	77	22.4
High (3 +)	‘High 2’ = First Warrant Officer to Captain and Grade 3 and 4 for civilians (code = 3) ‘High 1’ = Major and above and Grade 2 and below for civilian (code = 4) (Note: there were only a small number of cases and controls in ‘High 1’ category and therefore, this category was combined with’High 2’ in the analysis)	12	16	104	30.2
Medium (2)	Sergeant to Warrant officer and Civilian Grade 7–5 (code = 2)	37	49.3	135	39.2
Low (1)	Policeman to Corporal and Civilian Grade 8 or above (code = 1)	26	34.7	105	30.5
Employment Type					
Civilian	1	19	25.3	57	16.6
Officer	2	56	74.7	287	83.4
Education					
High 1 & High 2	Certificate and diploma or above (High 1) = 1 High school (High 2) = 2	26	34.7	172	50
Medium	Grade 7–11 (Medium) = 3	29	38.7	96	27.9
Low & No qualification	Grade 1–6 (Low) = 4 No Qualification but reads and writes (No qualification) = 5	20	26.6	76	22

Since the disability retirement outcome is binary and the study design is a matched case control study, the effects of each exposure and covariate on the risk of disability retirement were estimated using odds ratios and 95% confidence intervals calculated from fitting conditional logistic regression models as recommended by Rose and Laan [50] and Essebag et al. [51] using the matched set ID as the matching indicator for the analyses. A significance test for each exposure and covariate was calculated using a likelihood ratio test and the resulting p value is reported. Prior to fitting the conditional logistic regression models, the number of days of sickness absence for cases and controls were divided by 10 so that the resulting odds ratios are based on each additional 10 days of sickness absence.

When evaluating the effect of the average length of sickness absence spells on disability retirement an additional variable was created and added to the model. The variable was required so that the effect of average number of days was assessed only from information from participants who had one or more sickness absence spell. The variable was coded as 0 = those with no sickness absence spells and 1 = with sickness absence spells.

For each sickness absence measure, conditional logistic regression models were fitted first by adjusting for age, gender and work Administration only (the baseline matching criteria), and then adjusting additionally for demographic variables. Finally, adjustment for the corresponding sickness absence measure was also evaluated, (adjusting for the total number of days when examining risk of sickness absence spell on disability retirement and vice versa).

## Results

### Descriptive analysis

Table 3 describes the demographic characteristics of the cases and controls at the penultimate calendar year of the exposure period (baseline). Approximately, two thirds of the sample were aged 18–39 years and only one-fifth of the cases and controls were female. Cases were less likely to be married (59%) compared with controls (76% married).

In the cases and controls, 47% and 61% respectively were categorized as ‘field policing’ and the majority (75% (56/75) and 83% (287/344) respectively) were police officers. A balanced representation across employment grades was seen in the control group while 49% of cases were in ‘medium’ employment grades and 16% in the ‘high’ employment grade category. Regarding education, 35% and 50% of cases and controls respectively had Grade 12 (high school certificate) or above while 27% and 22% respectively had either ‘low’ or ‘no qualification’.

### Prediction disability retirement

#### Demographic Indicators

Table 4 shows the odds ratios for the association between demographic variables and disability retirement, estimated using conditional logistic regression. There was a statistically significant increase in the odds of disability retirement of more than two fold both in employees with medium, compared with high, employment grade and those with medium, compared with high, education levels. Increased odds of disability retirement were also seen in individuals with low, compared with high, employment grades (OR = 2.0, 95% CI = 0.96–4.41) and employees with low or no qualification compared with those with Grade 12 of education or above (OR = 1.77, 95% CI = 0.81–3.66) although these increases were statistically non-significant.

**Table 4:** Odds of disability retirement using demographic variables

Variable	Odds ratio for disability retirement			
	OR	95% CI	*P* value for heterogeneity	*P*
**Age**				
18–29				
30–39				
40–49				
50–59				
60 +				
**Gender**				
Female				
Male				
**Marital Status**				
Married	1			
Non-Married	**2.93**	**1.55–5.56**	0.001	< 0.001
**Occupation**				
Admin/ supportive	1			
Field Policing	**0.28**	**0.13–0.61**	0.01	0.001
Non-Field Policing	**0.43**	**0.19–0.96**		0.04
**Grade**				
High (3 +)	1			
Medium (2)	**2.39**	**1.18–4.84**	0.04	0.02
Low (1)	2	0.96–4.41		0.09
**Employment Type**				
Civilian	1			
Officer	0.57	0.27–1.18	0.14	0.13
**Education**				
High 1 &High 2	1			
Medium	**2.13**	**1.15–3.96**	0.05	0.02
Low & No qualification	1.77	0.81–3.66		0.15

When compared with their married counterparts, non-married police employees had a statistically significant increase in odds of disability retirement of almost three fold (OR = 2.93, 95% CI = 1.55–5.56). Non-field and field police officers, on the other hand, had significantly reduced odds of disability retirement compared with admin/supportive staff (OR = 0.43 and 0.28 with 95% CI = 0.19–0.96 and 0.13–0.61 respectively). Compared with civilian workers, officers had a lower odds of disability retirement, although this was not statistically significant (OR = 0.57, 95% CI = 0.27–1.18).

#### Sickness absence spells

Total number of sickness absence spells at baseline (OR = 1.17), final year (OR = 1.31) and total exposure period (OR = 1.18) was a statistically significant predictor of disability retirement. Apart from the number of spells of 1-3d in the penultimate calendar year, all other spell durations at each year of the exposure period significantly increased the odds of subsequent disability retirement (Table 5).

**Table 5:** Odds of disability retirement using type and period of spells of sickness absence

Type and period of spell	Total sample (*n *= 419)					Odds ratio for disability retirement		
	***Cases (n* = *75)***		***Controls (n* = *344)***					
	Mean	SD	Mean	SD	OR		95% CI	*P*
**Total number of all spells**								
Penultimate calendar year	4.1	0.5	2.3	0.2	**1.17**		**1.09–1.27**	** < *0.001***
Final calendar year	6.5	0.7	1.9	0.3	**1.31**		**1.21–1.41**	** < *0.001***
*Total exposure period*	10.7	1	4.2	0.4	**1.18**		**1.13–1.25**	** < *0.001***
**No. of spells of 1-3d**								
Penultimate calendar year	1.9	0.3	1.8	0.2	1.03		0.93–1.14	0.55
Final calendar year	2.7	0.4	1.5	0.2	**1.13**		**1.05–1.23**	** < *0.01***
*Total exposure period*	4.6	0.6	3.3	0.4	**1.06**		**1.01–1.12**	***0.02***
**No. of spells of 4-7d**								
Penultimate calendar year	0.7	0.1	0.2	0.4	**1.85**		**1.34–2.56**	** < *0.001***
Final calendar year	1	0.2	0.2	0.04	**2.08**		**1.52–2.84**	** < *0.001***
*Total exposure period*	1.6	0.3	0.4	0.1	**1.76**		**1.42–2.20**	** < *0.001***
**No. of spells of 8-28d**								
Penultimate calendar year	0.6	0.2	0.2	0.04	**1.59**		**1.19–2.12**	** < *0.01***
Final calendar year	1.4	0.2	0.1	0.04	**3.22**		**2.06–5.02**	** < *0.001***
*Total exposure period*	2	0.3	0.4	0.1	**1.96**		**1.54–2.49**	** < *0.001***
**No. of spells > 4 weeks**								
Penultimate calendar year	0.9	0.2	0.1	0.4	**3.36**		**2.08–5.41**	** < *0.001***
Final calendar year	1.5	0.2	0.1	0.1	**3.56**		**2.24–5.67**	** < *0.001***
*Total exposure period*	2.2	0.4	0.3	0.1	**2.47**		**1.79–3.40**	** < *0.001***

The conditional logistic regression results indicate that odds ratios for disability retirement for the total exposure period increased from 1.76 (95% CI = 1.42–2.20) for spells of 4-7d to 2.47 (95%CI = 1.79–3.40) for spells of > 4 weeks. Number of spells of sickness absence lasting > 4 weeks also resulted in the highest odds of disability retirement than other types of spells in the penultimate calendar year (OR = 3.36, 95% CI = 2.08–5.41) and in the final year prior to disability retirement (OR = 3.56, 95% CI = 2.24–5.67) and during the whole exposure period (OR = 2.47, 95%CI = 1.79–3.40).

#### Number of days of sickness absence

The odds of disability retirement for all sickness absence spells and for the average length of sickness absence spells are shown in Table 6. There were statistically significant increases in the odds of disability retirement at baseline (OR = 1.17), final year (OR = 1.32) and total exposure period (OR = 1.17).

**Table 6:** Odds of disability retirement using total and average days of sickness absence

Variable relating to number of days of sickness absence	Total sample (*n* = 419)				Odds ratio for disability retirement		
	***Cases (n* = *75)***		***Controls (n* = *344)***				
	Mean	SD	Mean	SD	OR	95% CI	*P*
**Total number of days of sickness absence for all spells**							
Penultimate calendar year	62.9	11.05	14.3	2.4	**1.17**	**1.10–1.25**	** < *0.001***
Final calendar year	103.2	10	13.2	5	**1.32**	**1.21–1.45**	** < *0.001***
*Total exposure period*	166.4	21.9	27.5	7	**1.17**	**1.11–1.23**	** < *0.001***
**Average length of sickness absence spells**							
Penultimate calendar year	17.9	3.1	6.9	1.2	**1.34**	**1.13–1.60**	** < *0.01***
Final calendar year	21.9	5.4	5.9	1	**2.05**	**1.51–2.77**	** < *0.001***
*Total exposure period*	19.9	3.5	6.9	1	**1.44**	**1.19–1.73**	** < *0.001***

As the number of days of sickness absence is expressed per 10 days of sick leave, this could be expressed as “after adjusting for matching variables (age, sex and work Administration), for each 10 additional days of sickness absence in the penultimate and final year prior to disability retirement, the odds of disability retirement increased by 17% and 32% respectively”. Finally, the results of Table 6 also showed that the average length of sickness absence spells during the exposure years was a significant predictor of disability retirement ((OR = 1.34, 95% CI = 1.13–1.60) for the penultimate calendar year, (OR = 2.05, 95%CI = 1.51–2.77) for the final year and (OR = 1.44, 95%CI = 1.19–1.73) for the entire exposure period).

#### Sickness absence adjusted for demographic variables

Table 7 compares the odds ratios of disability retirement at end of the exposure period for the matching variables with those obtained after additionally adjusting for all demographic variables (model b), namely, marital status, occupation, employment grade and type, and educational level. The odds ratios of disability retirement remained significantly raised for the total number of days of sickness absence and for the number of spells of sickness absence for all spell types, apart from spells lasting 1–3 days where the odds ratios became non-significant.

**Table 7:** Predicting disability retirement for sickness absence measure for matching (a) and adjusted (b) models

Total number of days of sickness absence/total number of sickness absence spell	OR (95% CI)	
	Model (a)	Model (b)
Total number of days of sickness absence	1.17 (1.11–1.23)	1.16 (1.10–1.23)
Total number of spells of sickness absence	1.18 (1.13–1.25)	1.17 (1.10–1.23)
Number of spells lasting 1-3d	1.06 (1.01–1.12)	1.02 (0.96–1.08)
Number of spells lasting 4-7d	1.76 (1.42–2.20)	1.77 (1.39–2.27)
Number of spells lasting 8-28d	1.96 (1.54–2.49)	1.95 (1.52–2.51)
Number of spells > 4 weeks	2.47 (1.79–3.40)	2.33 (1.67–3.24)

### Summary of findings

This study evaluated the relationship between sickness absence and disability retirement using a matched case–control study design. The study included 75 sets of disability retirement cases (75 cases and 344 controls) who retired between 2010 and 2012. Controls were selected using disability retirement cases details including age, gender and administration in which the disability retirement case at baseline (two years prior to retirement of the case). Sickness absence records for two consecutive years were then obtained from the electronic register starting from baseline year.

The relationship between sickness absence and the risk of disability retirement was evaluated using conditional logistic regressions. Three sickness absence measures were used which are the number of sickness absence spells, the number of days of sickness absence and the average length of sickness absence spells. Disability retirement cases had a mean number of spells of sickness absence of 10.7 compared with 4.2 mean spells for controls. During the two-year exposure period, 44% of cases had 11 spells or more compared with only 10% of controls. One third of disability retirement cases had 160d or more of sickness absence during the two-year exposure period while most of controls had 39 days of sickness absence or less during the two-year exposure period.

The total number of sickness absence spells was a significant predictor of disability retirement. Conditional logistic regression results indicated a gradual increase in the risk of disability retirement with the increase in the range of sickness absence spells. The total number of days of sickness absence and the average length of sickness absence spells at baseline, final year and total exposure period were also significant predictors of disability retirement.

The odds ratio of disability retirement at the end of the exposure period for each of the three sickness absence measures were compared using chi-squared values. The strongest association was seen for the total number of sickness absence spells followed by the total number of days of sickness absence and the average length of sickness absence spells.

Finally, the association between the total number of sickness absence spells and the total number of days of sickness absence for the two-year exposure period and the risk of disability retirement was then evaluated by adjusting additionally for demographic and corresponding sickness absence measure. The associations remained statistically significant when demographic variables or corresponding sickness absence measure were added.

## Discussion and conclusions

This study is one of very few studies that have examined the use of various sickness absence measures as risk markers for disability retirement. The number of spells and total number days of sickness absence for cases in comparison the control group is significantly high which presents a trend that substantiate the assumption of this study and aligns with results of several other studies [41, 52, 53] that sickness absence is a determinant of disability retirement among the police as well. Also, the average length of sickness absence spells during the penultimate and final year prior to retirement in disability retirement cases in this study was 18 and 22 days while controls had an average of 7 and 6 days.

This study also presents an alarming trend among adults in the policing profession. Over 60% of the cases of disability retirement was reported among adults between 18 to 40 years of age. While this age group can be the most employed in policing given their ability and endurance, this also points to larger health cost implications for countries such as the UAE where the average life expectancy at birth is 77.9 years [54]. This could add to the fiscal burden of countries to support its retirees. Although UAE reports its health expenditure to be 4.28% of its GDP in comparison to 10.15 in the United Kingdom and 16.77 in the United States [55], it shows an upward trend over the past years and hence attention to its critical security personnel and their health is important.

In general, the total number of sickness absence spells (regardless of spell duration) and for each spell duration (except for spells lasting 1–3 days), the total number of days of sickness absence for all spells and the average length of sickness absence spells were all significant predictors of sickness absence. Apart from spells lasting 1–3 days, the results remained statistically significant even after adjusting additionally for other demographic variables. One outcome that is clear and stands out is that higher the number of spells and number of days and more importantly the higher the number of spells of days more than four weeks (long-term spells), the possibility of disability retirement goes up by at least three times both in the penultimate and the final calendar year. While this aligns with previous studies [22, 23, 56] that report the impact of long-term absence, this study provides new insight to understand the absence behavior over two years. The implication of this is if a police officer request for long-term sickness leaves in higher frequency in a year, with one or more term of four weeks in year, immediate intervention is recommended to mitigate possibility of filing for disability retirement.

In conclusion, this study investigated the association of various measures of sickness absence on disability retirement in the police personnel in Abu Dhabi. The outcomes indicate a strong association of absence and retirement even after controlling for demographic variables. Thus there are many strengths of this study. First, the study matched cases to eligible controls with similar age, sex and same working Administration (at baseline). Sickness absence records were then collected by following cases and controls prospectively (from baseline until disability retirement was granted to cases). This has ensured that cases are matched with controls who had similar work exposures at the start of the study.

Secondly, although many of the previous studies utilized register-based data of sickness absence, one main strength of this study is the fact that Abu Dhabi Police implements a rigid system of recording sickness absence whereby all sickness absence spells regardless of duration are recorded in the Sickness Absence Human Resources System. This has allowed for accurate estimation of the association of short term sickness absence spells of 1–3 days with disability retirement. Thirdly, this study is one of very few to evaluate risk of disability retirement for different measures of sickness absence [3, 38] or average length of sickness absence spells [3, 10].

In addition, the evaluation of the sickness absence in this study was made using various measures of sickness absence, namely, number of days of sickness absence, number of spells of sickness absence and average length of sickness absence spells. This has allowed the results to be displayed for each exposure period separately and combined as well as all spells (regardless of spell duration) and for each spell duration separately. Although these three sickness absence measures predicted disability retirement, this study showed that the number of sickness absence spells and the number of days of sickness absence are equally good predictors of sickness absence while the average length of sickness absence spells had lower predictability of disability retirement than the latter two sickness absence measures.

Finally, controlling for age, gender and work Administration at baseline (during selection of controls) and additionally for other demographic variables and corresponding sickness absence measure could be considered as a strength of this study since only four of the previous studies incorporated adjustments in the analyses: Lund et al. [57] adjusted only for age, Kivimaki et al. [38] adjusted results for age, SES, town and other sickness absence measures, Labriola and Lund [52] and Ahola et al. [53] adjusted additionally for health behaviour, work factors and clinical factors (not valid for Lund et al. [57]). Future studies can control for these measures including age and gender to understand the relation of sickness absence measures to disability retirement in police and other occupational groups robustly.

As like other studies, this study is not without limitation. Firstly, the number of cases for the three years of the study is rather modest (*n* = 75). Although more cases can add to the significance of the relationship, Hennessy et al. [45], illustrated that the power of the case–control studies is impacted more by the ratio of case to control (1:4 or 5). Additionally, the cases are also limited by the availability of data (commencement starting 2010) and the sensitivity of the data (privacy concerns). Secondly, after identifying cases of disability retirement, controls were selected from the Administration where the case worked at baseline (two years prior to granting of disability retirement). As cases and controls may have changed work Administration after baseline, their work exposures may differ, however, controlling for such effects was not possible. The inability to control for other potential predictors of disability retirement such as medical diagnoses and organizational and occupational job factors are other limitations for this study.

Finally, comparing this study’s findings with others was difficult due to variation in the definitions used to classify spells into short and long term sickness absence spells. For example, Jensen et al. [56] defined long spells as more than 30 days of sick leave for upper limb disorder and 90 days or more of sickness absence for low back pain while Kivimaki et al. [38] recognized short term spells as 1–3 days and long term as more than three days of sick leave. Finally, differences between the findings of this study and other studies could be related to variations in sickness absence policies and regulations. In the UK a “fit to work” note must be obtained for sickness absence of more than 8 days [48] while the Abu Dhabi Police implement this procedure only for spells lasting four weeks or more.

Barring its limitations, this study has important implications for monitoring and mitigating disability retirement in Abu Dhabi Police. The disability retirement in police personnel in Abu Dhabi have a strong association with sickness absence irrespective of the length (total or average) or number of spell of sickness leave. This indicates the impact of physically more strenuous jobs [22] and high job demands [42] having an impact on long-term health effects of the police personnels. While, this study does not diagnose various health measures [2], this does indicate a need for intervention to reduce the possibility of disability retirement within this occupational group. Bramberg et al. [58] recommend applying a structured problem-solving process addressed through stepwise meetings between the line-managers and the employee. This could help identify early interventions and maintain regular communication through education and training with probable candidate of disability retirement.

## Data Availability

The datasets generated and/or analyzed during the current study are not publicly available due to privacy and the sensitivity of the data (as per UAE regulations).

## Footnotes

1 2019 and later years are not discussed as the cases may be skewed owing to COVID-19.
